# The Director of Charities and Correction in Philadelphia

**Published:** 1903-06

**Authors:** 


### The Director of Charities and Correction in Philadelphia.

IN PHILADELPHIA there has been announced an appointment, the principle of which might well be followed by many another city in this country with advantage to itself and credit to a profession, which has done and is daily doing more for humanity than any other class. The City of Brotherly Love has so far reformed in its civic side as to see wisdom in appointing Dr. Edward Martin as Director of Charities and Correction; and this step in the right direction is made by Mayor Weaver, a gentleman who has added glory enough to his administration by this one act to last him for all time, if he never did another really meritorious act during his term of office.

In Philadelphia, the Director of Charities and Correction controls the general municipal management of health and sanitation and there could not have been selected a more eminent scientist or a more fearless and honest man than Dr. Martin for the place which, by this appointment, has been removed beyond the grasp of the politicians who have done much to add shame to the municipal reputation of an otherwise delightful city. Affairs will be conducted in a radically different manner under Dr. Martin's guidance than they have ever been handled before in the history of the city; and as evidence of this fact it is only necessary to mention the names of the six gentlemen whom he invited to serve with him as an advisory board: Drs. S. Weir Mitchell, Hobart Amory Hare, J. William White, Charles Penrose, John H. Musser and J. M. Anders. The medical profession does not contain more eminent names than these and the fact of their acceptance of what will be a labor of love speaks well for their citizenship and their civic pride. It shows, too, that they will not pose as figureheads; it means they will bring to the aid of Dr. Martin in his work of purification, wide experience, earnestness of purpose and deep scientific knowledge, and that Philadelphia will be the most cleanly city in America.

Aside from the merit of the appointment of Dr. Martin and the public spirit shown by his associates there is still a more important significance to be attached to the event, and that is the example set by these eminent medical men in entering public life. There are too few physicians who take an active part in civic government in the United States. In Europe and in South America we find in important public office, in diplomacy, in statecraft, in education, in public health, the most prominent figures to be those of physicians; in this country the great majority appear to be willing to cease their efforts for public office when they secure appointment as postmortem examiner, or coroner, or physician to a public institution. The cessation of effort at the acquisition of these offices is neither praiseworthy nor elevating. The offices mentioned could not very well, in this part of the country at least, go to any but a physician. As a rule, coroner's offices are rank with inefficiency. This has been changed in Erie County and other sections are preparing to follow us in the work of improvement of service and morals in this office.

Buffalo has had a physician as mayor and in spite of party prejudices and irrespective of political soreness, which is bound to exist, none can gainsay that Dr. Diehl made an excellent and high-minded executive. That he made mistakes, is possible; but they were the innocent mistakes of one who is more of a gentleman than a pot-house politician and who served the people as he thought they should be served, honestly and to the best of his ability. He did what he thought was right and his administration was clean and firm in principle.

It has been said that physicians are too busy to go into politics. Rather let it be said that they are too honest as a body to mix up in the trickery of ward deals. They should not be too busy to give some of their time to the work of elevating the cities in which they live or the states of which they are citizens. Physicians by education and experience are versed in the intricacies of human nature. They are warm-hearted and sympathetic friends, but cold and calculating reasoners. That is the secret of the success of the continental and South American physician in public life. As a profession we are too timorous. Let us push forward the best men in medicine and the records they make in public life will lift up the younger men, and give them examples of a lofty public spirit which they will not hesitate to strive to emulate. And, incidentally, it will increase public respect for the profession as a whole.

---

The State Consumption hospital at Raybrook received a substantial appropriation from the last legislature, and, we are happy to learn, Governor Odell has approved the full amount granted. This is as it should be, and enables the trustees to proceed along lines that will place the State of New York where it belongs, in the front rank of commonwealths in dealing with this dread disease that annually sweeps away 13,000 of our inhabitants. The temptation is very strong for a governor to reduce many items in the annual appropriation bills, and very properly so, but in this instance Governor Odell has shown himself to be in touch with the most progressive spirit of preventive medicine, and deserves the thanks of every physician and all enlightened citizens.

---

The Journal cannot refrain making a brief reference in this issue to a notable example of decency of purpose on the part of the publishers of *Everybody's Magazine*, the June number of which has been placed on sale just as we are going to press. This magazine has changed hands and is now owned by two men who have spent years in magazine work. Heretofore it was the property of a big department house and, like the majority of magazines, was publishing everything in the way of advertising which was paid for, chief and most objectionable of which were the patent medicine advertisements. In the announcement of the policy of the magazine under the new management this statement is made:

It is our intention to insert only high-grade advertising in our magazine. . . . We shall exercise a scrupulous censorship over all the advertising pages. Various lines of patent medicine and other curative and objectionable advertising will be declined, even when offered by well-known and reliable firms. . . . We inherit a few contracts which we shall not accept after they have expired. Whether it is desirable to publish patent medicine, curative, and certain kinds of financial advertising in a magazine is a question for each publisher to decide. . . . . June is not a heavy month for advertising, but we have declined $900 worth of that kind of business—and we could use the money."

This is honest and refreshing and betokens a cleaner line of advertising. In a paper which has been prepared for the Journal on Medical Fakes and Fakirs, which was unavoidably crowded out of this issue, but which will be published next month, the question of disgusting and dishonest medical advertising in the monthly magazines is taken up at length; but the honesty and fearlessness of the new owners of *Everybody's Magazine* are worthy of especial mention at this time, as indicating that because a magazine is sold for ten cents it need not necessarily be a garbage mess so far as its advertising pages are concerned.

---

*Truth*, the new weekly newspaper which Mr. Mark S. Hubbell has given to the public in Buffalo, has already set the pace for the daily newspapers in many matters of local interest, and in his last issue he raises the standard of purity of advertising which can be followed by the daily newspapers without much monetary loss to themselves, and with much improvement in the tone of their business columns. Mr. Hubbell boldly declares against the fakes, fakirs and the criminal abortionists.

---

THE Farbenfabriken of Elberfeld Company, of New York, is forcing the issue all along the line in regard to drug substitution. There appears to be an organised attempt on the part of certain persons to supply druggists with bogus phenacetin, trional, aristol, and other Bayer products, the druggist being tempted by the statements that these goods are genuine, but contraband, and therefore can be sold at a much cheaper rate. It has been observed, however, that these bogus products which are sold in counterfeit boxes, are, for the most part, composed of acetanilid.

The company referred to is bringing action in the Federal courts against all persons whom it detects in the unlawful practice of substituting as related to any of the products which it manufactures or controls.

Recently the campaign against infringers was opened in Tennessee. Bills have been filed within the last few weeks against druggists in Memphis, Nashville, Chattanooga, Knoxville and Dyersburg, and in most of these cases the defendants have already thrown up their hands and asked for quarter. The remainder, it would seem from present indications, are likely to follow suit and settle upon the best terms they can secure, after having examined the record made in the United States court for Tennessee in the case which has just been prosecuted to a conclusion against J. T. Hampton, of Cleveland, Tenn.

It has been the policy of certain pharmaceutical journals, in order to shield unscrupulous druggists, to state that the efforts to suppress substitution are simply an attempt to gain free advertising for certain products in the medical journals. It is hardly necessary to say that such standard drugs as phenacetin, sulphonal, trional, and aristol require no advertising to make them known to the physician. The sole object is to compel the druggist to dispense these products when called for on a prescription, and not the bogus substitutes which he has been obtaining from illegitimate sources.

The house referred to is to be commended for the thoroughness with which it is pursuing the offenders, and it is to be hoped that the full penalties of the law will be meted out to those who are proven guilty.

THE Regents of the University of the State of New York, at their regular meeting, held at Albany, May 21, 1903, made appointments of state medical examiners as follows:

Representing the State Medical Society—Dr. William Warren Potter, Buffalo; Dr. William S. Ely, Rochester; Dr. Maurice J. Lewi, New York.

Representing the Homeopathic Medical Society—Dr. John M. Lee, Rochester; Dr. J. Willis Candee, Syracuse; Dr. George E. Gorham, Albany. Dr. William M. S. Fiske, Brooklyn, was appointed in the place of Dr. William Morris Butler, Brooklyn, resigned.

Representing the Eclectic Medical Society—Dr. L. H. Smith, Buffalo; Dr. O. W. Sutton, Bath; Dr. H. H. Nichols, Worcester.

---

GOVERNOR ODELL has signed Mr. Sherry's bill making it a misdemeanor to distribute samples of drugs or patent medicines in such a way that children may get them.

In the JOURNAL for April, 1903, page 691, we made reference to a fatal poisoning caused by house-to-house distribution of samples of medicines. This law is evidently aimed at such practices, which must now stop.

